# Unsupervised Network Analysis of the Plastic Supraoptic Nucleus Transcriptome Predicts Caprin2 Regulatory Interactions

**DOI:** 10.1523/ENEURO.0243-17.2017

**Published:** 2017-12-21

**Authors:** Su-Yi Loh, Thomas Jahans-Price, Michael P. Greenwood, Mingkwan Greenwood, See-Ziau Hoe, Agnieszka Konopacka, Colin Campbell, David Murphy, Charles C. T. Hindmarch

**Affiliations:** 1 Department of Physiology, Faculty of Medicine, University of Malaya, Kuala Lumpur 50603, Malaysia; 2 School of Clinical Sciences, University of Bristol, Bristol BS1 3NY, United Kingdom; 3 Department of Engineering Mathematics, University of Bristol, Bristol BS8 1UB, United Kingdom; 4 Queen’s Cardiopulmonary Unit (QCPU), Translational Institute of Medicine (TIME), Department of Medicine, Queen’s University, Kingston, Ontario, ON K7L 3N6 Canada

**Keywords:** *Caprin2*, functional plasticity, gene network, Glasso, supraoptic nucleus, transcriptome

## Abstract

The supraoptic nucleus (SON) is a group of neurons in the hypothalamus responsible for the synthesis and secretion of the peptide hormones vasopressin and oxytocin. Following physiological cues, such as dehydration, salt-loading and lactation, the SON undergoes a function related plasticity that we have previously described in the rat at the transcriptome level. Using the unsupervised graphical lasso (Glasso) algorithm, we reconstructed a putative network from 500 plastic SON genes in which genes are the nodes and the edges are the inferred interactions. The most active nodal gene identified within the network was *Caprin2*. *Caprin2* encodes an RNA-binding protein that we have previously shown to be vital for the functioning of osmoregulatory neuroendocrine neurons in the SON of the rat hypothalamus. To test the validity of the Glasso network, we either overexpressed or knocked down *Caprin2* transcripts in differentiated rat pheochromocytoma PC12 cells and showed that these manipulations had significant opposite effects on the levels of putative target mRNAs. These studies suggest that the predicative power of the Glasso algorithm within an *in vivo* system is accurate, and identifies biological targets that may be important to the functional plasticity of the SON.

## Significance Statement

The scale and complexity of transcriptome datasets makes the identification of suitable targets for physiologic studies a daunting problem. Using the unsupervised graphical lasso (Glasso) algorithm, we reconstructed a putative network from 500 plastic genes in the supraoptic nucleus (SON) of the hypothalamus. The most active nodal gene identified within the network encodes RNA-binding protein CAPRIN2. We tested the validity of the Glasso network by either overexpressing or knocking down *Caprin2* transcripts in differentiated rat pheochromocytoma cells and showed that these manipulations had opposite effects on the levels of putative target mRNAs. Our studies suggest that the predicative power of the Glasso algorithm can identify biological targets that may be important in a *Caprin2* gene network mediating functional plasticity in the SON.

## Introduction

The supraoptic nucleus (SON) of the mammalian hypothalamus is a central neuroendocrine integrative structure consisting of large magnocellular neurons (MCNs) whose axons project to the posterior lobe of the pituitary (PP; Burbach et al., 2001), a neurovascular interface through which the brain regulates peripheral organs to maintain homeostasis (Murphy et al., 2016). The SON is responsible for the synthesis of the neuropeptide hormones arginine vasopressin (AVP), which cleaved from its precursor propeptide *en route* from the SON to the PP (Murphy et al., 2016). On release, AVP travels through the blood stream to specific receptor targets located in the kidney where it promotes water reabsorption in the collecting duct (Breyer and Ando, 1994).

Lactation and dehydration evoke a dramatic remodelling of the SON (Hatton, 1997; Theodosis et al., 1998). A plethora of changes in morphology, electrical properties and biosynthetic and secretory activity have all been described (Sharman et al., 2004). For example, as a consequence of the depletion of pituitary stores that accompanies chronic osmotic stimulation, there is a need to synthesize more AVP. This starts with an increase in transcription (Murphy and Carter, 1990), which results in an increase in the abundance of both precursor hnRNAs (Kondo et al., 2004) and mature *AVP* mRNAs (Sherman et al., 1986). In addition, It has been demonstrated that the *AVP* mRNAs is subject to post-transcriptional modification in the form of an increase in the length of the 3’ poly(A) tail following dehydration (Carrazana et al., 1988; Zingg et al., 1988; Carter and Murphy, 1989; Murphy and Carter, 1990). Recently, microarrays have been used to document transcriptome-wide changes in gene expression in the SON of male rats subject to salt-loading and dehydration (Hindmarch et al., 2006; Greenwood et al., 2015), and in female rats in response to both dehydration and 11 d of lactation (Qiu et al., 2011), and it has been suggested that these changes are part of an organized response to maintaining homeostasis in a changing environment (Hindmarch et al., 2013).

The analysis pipeline for these, and indeed most microarray experiments, is one of normalization and statistical testing before the filtering according to some arbitrary cutoff, such as fold-change. The resulting list of transcripts can then either be mined manually to select well-regulated targets, or subjected to one of the many approaches to bioinformatic analysis according to gene function or pathway. While these strategies are well used and often return important findings, they are at odds with the unbiased philosophy behind the transcriptome-wide experiment, as they require that the target gene has already been described, typically in a tissue discrete from that under interrogation. To investigate new strategies for target prioritization, we have here employed network reconstruction/inference strategies to our SON microarray data in either the control state or the “plastic” state to test whether this is an effective strategy for the robust identification of important plastic genes in this tissue.

Network inference is a strategy whereby a network structure is estimated from transcriptome data; genes are the nodes of this network and the edges are the inferred interactions. Two main strategies exist when attempting to reconstruct gene networks. The most tractable approach uses supervised inference where a set of “known” links and non-links between genes are used as a training set to construct a classifier or decision function. This classifier can be subsequently used to estimate links or non-links between further nodes in a network. In the second approach, unsupervised inference is used and the network structure is assumed only from the transcriptome data presented and thus the inference problem is harder. Here, we use the unsupervised graphical lasso (Glasso) algorithm (Friedman et al., 2008) to generate a putative gene network (see Materials and Methods for a full description). Gene features in Glasso are viewed as nodes in a network that predicts stable and reproducible dependencies between these nodes. To validate the results from the Glasso algorithm, we inferred a second network model based on the matrix of Pearson correlation coefficients between nodes.

From the derived Glasso network, we identified a hub nodal gene, *Caprin2*, that encodes an RNA-binding protein that we have previously identified as being important in the central osmotic defense response (Konopacka et al., 2015). To test this prediction from the Glasso algorithm, we perturbed the network *in vitro* by manipulating *Caprin2* expression. We show that the predicative power of the Glasso algorithm is accurate and identifies genes that may be important in biological transitions.

## Materials and Methods

### Microarray data preparation

We re-mined raw data taken from analysis of 29 Affymetrix 230 2.0 microarrays that represented animals in either the naïve state (*n* = 4 female and *n* = 5 male) or the plastic state (*n* = 5 dehydration-male, *n* = 5 lactation-female, *n* = 5 dehydrated-female, *n* = 5 salt-loaded-male; Hindmarch et al., 2006; Qiu et al., 2011; Greenwood et al., 2015). NCBI Accession numbers (incorporating controls): male dehydration, GSE3110; male salt loading, GSE65663; female dehydration and lactation, GSE30733. We note that the biological importance of salt and water balance, especially after challenge, ensures small standard deviations (10%) in the data (Hindmarch et al., 2013). Validation using quantitative reverse transcription PCR (qRT-PCR; Hindmarch et al., 2006; Qiu et al., 2011; Greenwood et al., 2015; Fig. 3) has shown that the microarray data are very robust and reliable. We note that the genes encoding the two major neuropeptide products of the SON, *AVP* and *oxytocin*, are not represented in our differentially expressed gene list. The expression level of both these important peptides was so high within the control data that the signal was saturated, precluding any detection of upregulation following dehydration, lactation or salt loading (Hindmarch et al., 2006).

**Figure 1.**
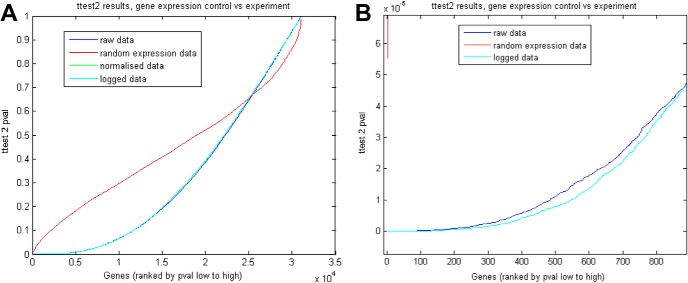
***A***, Microarray data *t* test, control versus experiment, genes ranked by ttest2 *p* value low to high. Raw data, normalized data, and logged data all gave comparable results. Randomly generated data gave higher *p* values for over half the gene pairs. Above the point of curve intersection microarray data stopped providing information, as significance was equivalent to randomly generated data. ***B***, Zoomed in version of ***A***, showing that the microarray data generated far lower *p* values compared to the random data. The lowest *p* value from randomly generated data were 1.5 × 10^−5^. The figure shows that the top 500 genes are under 1 × 10^−5^.

Initially trying to run Glasso on the entire SON microarray dataset of 31042 genes was prohibitive as a covariance matrix of 31042 × 31042 genes was too large to be stored in memory in R. Also, it is worth noting that many genes, even if well correlated with each other, were of lower interest as they are not necessarily strongly up or down regulated from control to experimental state. Therefore, it was necessary to reduce the size of the datasets to a manageable subset that only included potentially interesting genes, those that were significantly regulated between naïve and plastic states. To avoid the false discovery rate inherent in comparisons with sets of large numbers, we used a two-sample *t* test, comparing our experimental data against sets of 29 randomly generated matrices (using a Gaussian random deviate) of the same dimensionality (31042 × 31042; Fig. 1*A*). We performed a set of 10 such comparisons, against different sets of randomly generated matrices, and the most significant *t* test *p* values from this study were of order 10^−5^, the most significant *p* value from a comparison was 1.5 × 10^−5^ and the least significant was 6 × 10^−5^. When we applied this cutoff to the microarray data, the *t* test returned >500 gene pairs with higher significance (Fig. 1*B*) with *p* values up to 1 × 10^−15^. All data were run on raw, normalized and logged data for comparison. To establish the false discovery rate, and the level of significance that should be applied to relieve the effects of false discovery, we ran a *t* test on a randomly generated set of 29 (31042 × 31042) matrices (the same size as the experimental data matrices). We then applied these thresholds to our raw, normalized and logged data for comparison between these types.

### Network inference using the Glasso algorithm

The Glasso algorithm is based on determining an estimated precision matrix. This precision matrix is the inverse of the estimated sample covariance matrix, derived from data. For two features, a zero component of the precision matrix would mean the corresponding variance is infinite, i.e., that the correlation is uniform and infinitely spread, and the features are therefore totally uncorrelated. In our case these features can be viewed as nodes, representing genes or expression sites generally, in a network and the method is determining dependencies of expression levels. With the Glasso algorithm a penalty term is used to force components of the estimated precision matrix toward zero. Those components of the precision matrix which remain non-zero correspond to the highest interdependencies between nodes (e.g., genes) in the network.

The Glasso algorithm has various advantages over other algorithms for network inference. It is one of the fastest network inference algorithms available, running 30-4000 times faster than its competitors (Friedman et al., 2008). It can manage large amounts of data, running on 1000 × 1000 matrices in around a minute and the use of an L1 (Lasso) penalty term to enforce sparsity enables few key functional links to be inferred from large amounts of data. This fitted our problem well as we wish to obtain a few high-probability links to test experimentally from a large amount of data.

Glasso estimates a sparse undirected graph by applying an L1 penalty to the estimation of an inverse covariance matrix. The model assumes that data (in this case, gene expression data) is continuous and that the observations comes from a multivariate Gaussian distribution, that has mean μ and covariance matrix Σ. This covariance matrix encodes the extent that gene expression values are correlated and therefore its components give a measure of strength of correlation. As such the matrix is symmetrical along its diagonal. An estimation of the inverse of the covariance matrix, called the estimated precision matrix, is used to infer potential links.

To determine those features which are most interdependent Glasso uses a penalty term in the algorithm to force components of the estimated precision matrix to zero. A parameter ρ, prefacing this penalty term, controls the extent of sparsity in the estimated network. Those remaining components of the precision matrix which remain non-zero correspond to the highest interdependencies among nodes (genes) in the network. Let *S* be the sample covariance matrix and *S*^−1^ be its inverse. Let Γ be the estimated precision matrix. The objective function of the Glasso algorithm has two terms, which are optimized via an algorithm. The first term is of the form *KL*[*N*(0, Γ), *N*(0, *S*^−1^)], where *KL* is the Kullback-Leibler divergence, a measure of similarity of distributions, and *N*(0, *M*) is a Gaussian distribution with a zero mean vector and precision matrix *M*. Minimization of this term would therefore enhance the similarity of the estimated precision matrix Γ to the inverse sample covariance matrix. The second term in the objective function is ρ|| Γ ||_1_ where || Γ ||_1_ is the sum of the absolute values in Γ. Minimization of this term will force the values of the components within Γ to zero. Effectively then, the algorithm tries to move Γ as closely as possible toward *S*^−1^ subject to the influence of the second term, which is trying to force component values within the matrix Γ toward zero, the relative influence of these two terms controlled by ρ. Those remaining components of the precision matrix which remain non-zero correspond to the highest interdependencies among nodes in the network.

The sample covariance matrix becomes singular when the dimension of the matrix is much larger than the sample size. For this reason, to model large covariance matrices, the usual approximation is via a sparse estimation matrix, with most entries zero (Bickel and Levina, 2008). This is the route taken by the Glasso algorithm, in which optimization of a norm-1 penalized maximum likelihood leads to a sparse estimation of the precision matrix (Friedman et al., 2008). To further understand this issue we used the F07AGF routine of the Numerical Algorithms Group (NAG) Library (Mark 24) to determine the condition number μ of the matrices used in our study. Approximately, if we determine that the condition number μ to be of order *1.0E+k* then *k* digits of numerical accuracy would be lost due to accumulated arithmetical loss of precision (Cheney and Kincaid, 2007). For our dataset with 500 features (representing genes), the NAG routine gives a condition number of *0.69E+15* for the sample covariance matrix. Hence, the data-derived covariance matrix itself is close to being rank-deficient (though no covariance matrix inversion or other data manipulations are required by the method). For the least sparse approximation we used in our numerical experiments, the estimated condition number of the sparse precision matrix was *7.17E+5*, suggesting numerical accuracy was satisfactory for the given machine precision. However, this issue of the condition number required us to use the subset of 500 genes, rather than the full set of genes.

Using a sample covariance matrix derived from the data, Glasso iteratively derives an estimated inverse covariance matrix (precision matrix). The algorithm uses an L1-norm penalty to drive elements in this precision matrix toward 0. This penalty therefore determines the sparsity of the estimated precision matrix and hence the sparsity of the network of assumed dependencies. This penalty term is prefaced by a parameter ρ and the higher the value of ρ, the greater the sparsity. To find an appropriate value for ρ we chose the known links and non-links of a well-studied pathway, to give an indication of the correct network sparsity, and best choice for ρ. The ERK pathway data evaluated (Sachs et al., 2002) consists of 11 proteins and 11943 observations of their expression levels from 12 perturbation experiments. The ERK pathway is very well studied and a full network of directional links is known for the network. We downloaded the Glasso software (from http://statweb.stanford.edu/∼tibs/glasso/) and applied it to this dataset. Setting the Glasso sparsity parameter to ρ = 4 gave those links and non-links which have been found in the ERK pathway (Sachs et al., 2002).

In addition to running Glasso on the top 500 differentially expressed genes, a Pearson correlation measure was also derived from the data. The genes pairings returned by Glasso were in the top most highly correlated pairings as determined by the Pearson correlation coefficient.

### Animal experiment

All experiments were performed under a Home Office United Kingdom license held under, and in strict accordance with, the provisions of the United Kingdom Animals (Scientific Procedures) Act (1986); they were also approved by the local Animal Welfare and Ethical Review Board. Twelve-week-old male SD Rats (Harlan) were given access to standard laboratory rat chow and water ad libitum for one week to acclimatize to laboratory conditions, following which half the animals were completely deprived of drinking water for 72 h (dehydration), which elicits a reliable and consistent increase in plasma osmolality with limited weight loss (Greenwood et al., 2015). Animals were killed by stunning and then decapitated with a small animal guillotine (Harvard Apparatus). The brain was quickly extracted and placed on aluminum foil and frozen with dry ice. Brains were stored at -80°C.

### Functional validation of the Glasso-derived gene network

The rat adrenal pheochromocytoma cell line (PC12) was grown complete DMEM (Sigma D6546) containing 10% (v/v) horse serum (HS; Gibco 16050), 5% (v/v) heat-inactivated fetal bovine serum (Gibco 10500), 1% (v/v) penicillin/streptomycin solution (Pen Strep; Gibco 15140), and 2 mM L-glutamine (Gibco 25030). Cells were cultured onto tissue culture flasks precoated with 40 µg/ml rat-tail Type I collagen (Type VII, C8897, Sigma Aldrich) and maintained at 37°C in a humidified incubator with 5% (v/v) CO_2_. On reaching confluence, cells were passaged and plated onto collagen-coated 12-well tissue culture plates with complete DMEM as described above. Differentiation of PC12 cells was initiated by replacing the medium with serum-free DMEM containing 0.25% (w/v) bovine serum albumin (Sigma A17906), 1% (v/v) HS, 1% (w/v) Pen Strep solution, and 50 ng/ml nerve growth factor (Life Technologies13257-019). Medium and NGF were replaced every 2 d. At day 4 of NGF treatment, *Caprin2* shRNA lentivirus was added to the medium, with a scrambled shRNA lentivirus being used as a control. Lentiviral vectors containing shRNA of *Caprin2* and scrambled shRNA control were produced as previously described (Konopacka et al., 2015). For overexpression studies of *Caprin2* in PC12 cells, an adenovirus expressing full-length *Caprin2* cDNA (Konopacka et al., 2015) was used, with an *eGFP*-expressing virus as a control. Production of adenoviral vectors has been described (Greenwood et al., 2015).

### qRT-PCR

Tissue from SON was sampled using a cryostat, taking 60-μm coronal slices, and the SON was punched using a 0.5-mm tissue punch (Interfocus). Twelve punches were taken from the left and right SON (24 total per brain) and stored on dry ice in 1.5-ml tubes. Total RNA was extracted from punched samples by combining TRIzol reagent with Qiagens RNeasy kit protocols (Qiagen). The punched samples were removed from dry ice and rapidly resuspended, by vortexing, in 1-ml TRIzol reagent. Following TRIzol phase separation with chloroform, 350 μl of the upper aqueous phase was removed, mixed with 350-μl 70% (v/v) ethanol and applied to RNeasy columns. The remaining steps were performed as recommended by the manufacturer. For cell studies, cells were collected and lysed with 350-μl TRIzol reagent on day 7 after NGF treatment (3 d after virus transduction). RNA extraction was performed using Direct-Zol RNA Mini Prep Kit (Zymo Research) following the manufacturer’s instructions. RNA was converted to cDNA using QuantiTect Reverse Transcription kit (Qiagen). Primers for qPCR were designed using the NCBI PrimerBLAST tool (http://www.ncbi.nlm.nih.gov/tools/primer-blast/; Table 1). Note that the primer set for *Hbb* used for the analysis of Sprague Dawley (SD) SON RNA (derived from sequence BI287300) did not detect PC12 transcripts, presumably due to that cell line being derived from a different strain of rat. We therefore used primers designed to detect all rat Hbb alleles. PCRs were performed on ABI7500 or StepOnePlus Real-time PCR Systems (Applied Biosytem) using FastStart Universal SYBR Green Master Mix (Roche). The housekeeping gene *Rpl19* was used to normalize expression from targets and to calculate $2^{-\Delta \Delta C_{T}}$ (Livak and Schmittgen, 2001) to analyze the relative change in gene expression.

**Table 1.** List of primers used for qRT-PCR

Gene	GenBank	Primers
*Caprin2*	AI412606	Forward CAGGGTTAAGTGCAAGCGAT Reverse CTGGTGGTTGACTGGTTGAG
*Atf4*	NM_024403	QuantiTect primer assay; Rn_Atf4_1_SG: QT00366233
*Hbb*	BI287300 NM_033234	Forward GCCCAAAGGCCTTCATCATT Reverse CCCCCTTTCCTGCTTGTCTA QuantiTect primer assay; Rn_Hbb_1_SG: QT00394107
*Opn3*	BI289640	Forward CGACTGACAGGGACTCATCA Reverse ATGGGACAGGCCAAAGAAGA
*Ran*	NM_053439	Forward CGCGTGTGCCACCTTATTTA Reverse CCAAACAGCTAAATATGCAAGTCC
*Pcp4*	NM_013002	Forward TCAGGAGATAATGATGGGCAGA Reverse CCCCACTAGGACTGTGATCC
*Igfbp2*	NM_013122	Forward AACCTGTACCTCCGTTCCTG Reverse CCCAAGCCTGTACCCAGTAT
*Pdyn*	BF412731	Forward CCAGCCCCATCTCCTTAACT Reverse AGACTGTTCCCCCTCGGTAT
*Ap1s2*	AI045228	Forward ACCAATGCCACTTTGCTTCA Reverse CTGCCTAGTCGTCGGAAGTC
*Atp1a2*	NM_012505	Forward GGATCCTCCTGGTGACCTTT Reverse CTGTTTCTTCTTGCCACCCC
*Hmgn2*	BM391736	Forward AGGATGTCTCTCCTGGAAGC Reverse TTGTTAGCACACGGAACACTT
*Gja1*	AI411352	Forward GCACTGTTGAAACCTCCCTC Reverse TGACGAGCAACTTGGATGTTT
*Hba1*	AI179404	Forward AATCTTCCCCCAGCAGTTCTT Reverse CACTATAGGGAATTTGGCCCTC
*EST*	AI577319	Forward ATCTCCAAGGTGGTGGGAAG Reverse AAACTGGGTGTGGATCCTGT
*Rpl19*	NM_031103.1	Forward GCGTCTGCAGCCATGAGTA Reverse TGGCATTGGCGATTTCGTTG

### Statistical tests

Statistical differences between two experimental groups were evaluated using independent-sample unpaired Student’s *t* tests; *p* < 0.05 was considered significant.

## Results

## Significance testing of microarray data

Transcriptome datasets were derived from Affymetrix oligonucleotide array analysis of that the SD rat SON subject to three physiologic transitions that elicit functional plasticity, namely dehydration in both males (Hindmarch et al., 2006) and females (Qiu et al., 2011), salt-loading in males (Greenwood et al., 2015), and lactation in females (Qiu et al., 2011). To avoid the false discovery inherent in large comparisons, we employed a two-sample *t* test with a 31042 × 29 matrix of randomly generated data (see Methods) and established an appropriate significance threshold of *n* × 10^−5^. We selected the top 500 genes that were regulated in the SON between naïve and plastic states that satisfied the significance threshold (all *p* values < 1 × 10^−15^) and used these to establish the 500 × 500 covariance matrix computed and solved by Glasso.

### Network reconstruction using Glasso and Pearson

**Figure 2.**
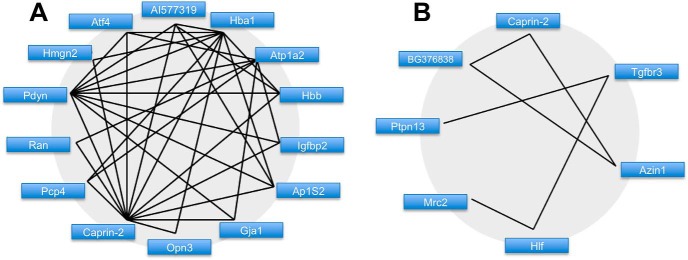
Putative gene networks derived from SON transcriptome data. ***A***, The Glasso-derived network. Undirected links are solid black lines. Genes are represented by short names. ***B***, The Pearson correlation-derived network. Undirected links are solid black lines.Genes are represented by short names.

Running Glasso with a L1 penalty parameter of 30 and ignoring links below a threshold of 0.0001 yielded 28 unique genes (Table 2) engaged in 48 links (Table 3). Pearson correlation returned 47 unique genes (Table 4) engaged in 32 bidirectional links with a correlation measure over 0.95 between (Table 5). We filtered these results to find those genes which had a high fan-out in terms of connectivity to other genes. We established subsets of genes with either a fan-out of more than one link, or at least one link to another gene with a fan-out greater than one. This resulted in networks of 14 genes from the Glasso reconstruction (Fig. 2*A*) and seven genes from the Pearson correlation (Fig. 2*B*). Only one gene satisfied these requirements in both networks; *Caprin2* was ranked 50th overall in the *t* test.

**Table 2.** List of unique genes in the Glasso network

1367576_at	S41066	*Gpx1*
1367590_at	NM_053439	*Ran*
1367624_at	NM_024403	*Atf4*
1367648_at	NM_013122	*Igfbp2*
1367681_at	NM_022523	*CD151*
1367887_at	NM_017024	*Lcat*
1368145_at	NM_013002	*Pcp4*
1368170_at	NM_024371	*Slc6a1*
1368565_at	NM_019225	*Slc1a3*
1370172_at	AA892254	*Sod2*
1370240_x_at	AI179404	*Hba1*
1370442_at	U25684	*Tmsbl1*
1371245_a_at	BI287300	*Hbb*
1371352_at	BM391736	*Hmgn2*
1372002_at	AI411352	*Gja1*
1373260_at	AI412606	*Caprin2*
1375856_at	AI102258	*EST*
1386911_at	NM_012505	*Atp1a2*
1388608_x_at	AI577319	EST
1388795_at	AI101500	EST
1389586_at	BE107169	EST
1398888_at	AI408819	*H3f3b*
1383294_at	BF412731	*Pdyn*
1393263_at	AW522530	*Snhg11*
1393373_at	BI289640	*Opn3*
1394940_at	BI294811	*Fam46a*
1395249_at	BF400750	*Snhg11*
1398616_at	AI045228	*Ap1s2*

Column 1, Affymetrix probe ID; column 2, GenBank accession number; column 3, current gene ID.

**Table 3.** Glasso pairs

1383294_at	BF412731	1367624_at	NM_024403
1373260_at	AI412606	1367624_at	NM_024403
1386911_at	NM_012505	1367624_at	NM_024403
1370240_x_at	AI179404	1367624_at	NM_024403
1373260_at	AI412606	1371352_at	BM391736
1370240_x_at	AI179404	1371352_at	BM391736
1367624_at	NM_024403	1383294_at	BF412731
1373260_at	AI412606	1383294_at	BF412731
1372002_at	AI411352	1383294_at	BF412731
1398616_at	AI045228	1383294_at	BF412731
1367648_at	NM_013122	1383294_at	BF412731
1371245_a_at	BI287300	1383294_at	BF412731
1386911_at	NM_012505	1383294_at	BF412731
1370240_x_at	AI179404	1383294_at	BF412731
1388608_x_at	AI577319	1383294_at	BF412731
1373260_at	AI412606	1367590_at	NM_053439
1386911_at	NM_012505	1367590_at	NM_053439
1373260_at	AI412606	1368145_at	NM_013002
1386911_at	NM_012505	1368145_at	NM_013002
1370240_x_at	AI179404	1368145_at	NM_013002
1367624_at	NM_024403	1373260_at	AI412606
1371352_at	BM391736	1373260_at	AI412606
1383294_at	BF412731	1373260_at	AI412606
1367590_at	NM_053439	1373260_at	AI412606
1368145_at	NM_013002	1373260_at	AI412606
1367887_at	NM_017024	1373260_at	AI412606
1393373_at	BI289640	1373260_at	AI412606
1389586_at	BE107169	1373260_at	AI412606
1394940_at	BI294811	1373260_at	AI412606
1398888_at	AI408819	1373260_at	AI412606
1370442_at	U25684	1373260_at	AI412606
1367576_at	S41066	1373260_at	AI412606
1372002_at	AI411352	1373260_at	AI412606
1398616_at	AI045228	1373260_at	AI412606
1367648_at	NM_013122	1373260_at	AI412606
1395249_at	BF400750	1373260_at	AI412606
1371245_a_at	BI287300	1373260_at	AI412606
1370172_at	AA892254	1373260_at	AI412606
1370240_x_at	AI179404	1373260_at	AI412606
1388608_x_at	AI577319	1373260_at	AI412606
1388795_at	AI101500	1373260_at	AI412606
1373260_at	AI412606	1367887_at	NM_017024
1373260_at	AI412606	1393373_at	BI289640
1370240_x_at	AI179404	1393373_at	BI289640
1373260_at	AI412606	1389586_at	BE107169
1373260_at	AI412606	1394940_at	BI294811
1373260_at	AI412606	1398888_at	AI408819
1373260_at	AI412606	1370442_at	U25684
1386911_at	NM_012505	1368565_at	NM_019225
1373260_at	AI412606	1367576_at	S41066
1370240_x_at	AI179404	1367681_at	NM_022523
1383294_at	BF412731	1372002_at	AI411352
1373260_at	AI412606	1372002_at	AI411352
1386911_at	NM_012505	1372002_at	AI411352
1386911_at	NM_012505	1368170_at	NM_024371
1383294_at	BF412731	1398616_at	AI045228
1373260_at	AI412606	1398616_at	AI045228
1370240_x_at	AI179404	1398616_at	AI045228
1388608_x_at	AI577319	1398616_at	AI045228
1383294_at	BF412731	1367648_at	NM_013122
1373260_at	AI412606	1367648_at	NM_013122
1386911_at	NM_012505	1367648_at	NM_013122
1370240_x_at	AI179404	1393263_at	AW522530
1373260_at	AI412606	1395249_at	BF400750
1383294_at	BF412731	1371245_a_at	BI287300
1373260_at	AI412606	1371245_a_at	BI287300
1370240_x_at	AI179404	1371245_a_at	BI287300
1388608_x_at	AI577319	1371245_a_at	BI287300
1386911_at	NM_012505	1375856_at	AI102258
1373260_at	AI412606	1370172_at	AA892254
1367624_at	NM_024403	1386911_at	NM_012505
1383294_at	BF412731	1386911_at	NM_012505
1367590_at	NM_053439	1386911_at	NM_012505
1368145_at	NM_013002	1386911_at	NM_012505
1368565_at	NM_019225	1386911_at	NM_012505
1372002_at	AI411352	1386911_at	NM_012505
1368170_at	NM_024371	1386911_at	NM_012505
1367648_at	NM_013122	1386911_at	NM_012505
1375856_at	AI102258	1386911_at	NM_012505
1367624_at	NM_024403	1370240_x_at	AI179404
1371352_at	BM391736	1370240_x_at	AI179404
1383294_at	BF412731	1370240_x_at	AI179404
1368145_at	NM_013002	1370240_x_at	AI179404
1373260_at	AI412606	1370240_x_at	AI179404
1393373_at	BI289640	1370240_x_at	AI179404
1367681_at	NM_022523	1370240_x_at	AI179404
1398616_at	AI045228	1370240_x_at	AI179404
1393263_at	AW522530	1370240_x_at	AI179404
1371245_a_at	BI287300	1370240_x_at	AI179404
1388608_x_at	AI577319	1370240_x_at	AI179404
1383294_at	BF412731	1388608_x_at	AI577319
1373260_at	AI412606	1388608_x_at	AI577319
1398616_at	AI045228	1388608_x_at	AI577319
1371245_a_at	BI287300	1388608_x_at	AI577319
1370240_x_at	AI179404	1388608_x_at	AI577319
1373260_at	AI412606	1388795_at	AI101500

List of the pairs which retained a covariance value >0.0001, i.e., potential links. Columns 1 (Affymetrix probe ID) and 2 (GenBank accession number) are the first gene, columns 3 (Affymetrix probe ID) and 4 (GenBank accession number) are the second gene. Note that the list contains duplicates, so although it is 96 long, there are 48 links.

**Table 4.** List of unique genes in the Pearson network

1367624_at	NM_024403	*Atf4*
1367648_at	NM_013122	*Igfbp2*
1367654_at	NM_031819	*Fat1*
1367660_at	NM_024162	*Fabp2*
1368170_at	NM_024371	*Slc6a1*
1368559_at	NM_017091	*Pcsk1*
1370030_at	NM_017305	*Gclm*
1370240_x_at	AI179404	*Hba1*
1370442_at	U25684	*Tmsbl1*
1370575_a_at	D50734	*Azin1*
1371433_at	BM384999	EST
1372754_at	BG666424	*Appl2*
1373092_at	BE109587	*Tgfbr3*
1373260_at	AI412606	*Caprin2*
1373380_at	AI169085	EST
1373699_at	BM391164	EST
1373870_at	BE110630	*Fam98a*
1374004_at	BM387902	*Prepl*
1374709_at	AI406795	*Hlf*
1374812_at	AA818197	*Ptpn13*
1374941_at	BF397951	EST
1375856_at	AI102258	EST
1375964_at	BF282282	*Psph*
1376836_at	BF419655	EST
1387037_at	AF022247	*Cubn*
1388145_at	BM390128	*Tnxa-psq*
1388608_x_at	AI577319	*Hba1*
1388770_at	BI275670	*Ufm1*
1388795_at	AI101500	EST
1389020_at	BM389149	EST
1389135_at	AW140637	*Ctps2*
1398348_at	AA945604	EST
1377725_at	AI575322	EST
1378320_at	BG373845	*Rlbp1*
1379566_at	AW527929	*Rbm11*
1379900_at	AI043697	*Aldh5a1*
1382008_at	AI044348	*Rnls*
1382021_at	AA850650	*Pkd2*
1382905_at	AI102514	*Mrc2*
1383413_at	AW531481	*Hhat1*
1391923_at	BG376838	EST
1392108_at	BF390648	*RM2*
1393165_at	BG377684	*Tmem206*
1393373_at	BI289640	*Opn3*
1393837_at	AI145227	EST
1394029_at	BF283049	*Vma21*
1398616_at	AI045228	*Ap1s2*

Column 1, Affymetrix probe ID; column 2, GenBank accession number; column 3, current gene ID.

**Table 5.** Pearson pairs

1367624_at	NM_024403	1374941_at	BF397951
1374941_at	BF397951	1367624_at	NM_024403
1391923_at	BG376838	1373260_at	AI412606
1398616_at	AI045228	1373260_at	AI412606
1370575_a_at	D50734	1373260_at	AI412606
1389135_at	AW140637	1373260_at	AI412606
1382008_at	AI044348	1388770_at	BI275670
1383413_at	AW531481	1377725_at	AI575322
1388770_at	BI275670	1382008_at	AI044348
1370575_a_at	D50734	1393165_at	BG377684
1393837_at	AI145227	1393373_at	BI289640
1373260_at	AI412606	1391923_at	BG376838
1379566_at	AW527929	1391923_at	BG376838
1373380_at	AI169085	1391923_at	BG376838
1370575_a_at	D50734	1391923_at	BG376838
1377725_at	AI575322	1383413_at	AW531481
1382021_at	AA850650	1367654_at	NM_031819
1372754_at	BG666424	1367654_at	NM_031819
1393373_at	BI289640	1393837_at	AI145227
1367660_at	NM_024162	1373870_at	BE110630
1367660_at	NM_024162	1370442_at	U25684
1376836_at	BF419655	1375964_at	BF282282
1394029_at	BF283049	1370030_at	NM_017305
1398348_at	AA945604	1392108_at	BF390648
1374709_at	AI406795	1389020_at	BM389149
1387037_at	AF022247	1374812_at	AA818197
1373092_at	BE109587	1374812_at	AA818197
1370030_at	NM_017305	1394029_at	BF283049
1374812_at	AA818197	1387037_at	AF022247
1373870_at	BE110630	1367660_at	NM_024162
1370442_at	U25684	1367660_at	NM_024162
1391923_at	BG376838	1379566_at	AW527929
1375856_at	AI102258	1368170_at	NM_024371
1368559_at	NM_017091	1374004_at	BM387902
1391923_at	BG376838	1373380_at	AI169085
1374709_at	AI406795	1382905_at	AI102514
1371433_at	BM384999	1382905_at	AI102514
1374004_at	BM387902	1368559_at	NM_017091
1373260_at	AI412606	1398616_at	AI045228
1375964_at	BF282282	1376836_at	BF419655
1388145_at	BM390128	1367648_at	NM_013122
1373699_at	BM391164	1367648_at	NM_013122
1389020_at	BM389149	1374709_at	AI406795
1382905_at	AI102514	1374709_at	AI406795
1373092_at	BE109587	1374709_at	AI406795
1367648_at	NM_013122	1388145_at	BM390128
1373260_at	AI412606	1370575_a_at	D50734
1393165_at	BG377684	1370575_a_at	D50734
1391923_at	BG376838	1370575_a_at	D50734
1392108_at	BF390648	1398348_at	AA945604
1367648_at	NM_013122	1373699_at	BM391164
1382905_at	AI102514	1371433_at	BM384999
1375856_at	AI102258	1379900_at	AI043697
1368170_at	NM_024371	1375856_at	AI102258
1379900_at	AI043697	1375856_at	AI102258
1373260_at	AI412606	1389135_at	AW140637
1374812_at	AA818197	1373092_at	BE109587
1374709_at	AI406795	1373092_at	BE109587
1367654_at	NM_031819	1382021_at	AA850650
1367654_at	NM_031819	1372754_at	BG666424
1388795_at	AI101500	1378320_at	BG373845
1388608_x_at	AI577319	1370240_x_at	AI179404
1370240_x_at	AI179404	1388608_x_at	AI577319
1378320_at	BG373845	1388795_at	AI101500

Pearson correlated pairs with a value >0.95. Columns 1 (Affymetrix probe ID) and 2 (GenBank accession number) are the first gene, columns 3 (Affymetrix probe ID) and 4 (GenBank accession number) are the second gene. Note that the list contains duplicates, so although it is 64 long, there are 32 links.

### qRT-PCR validation

**Figure 3.**
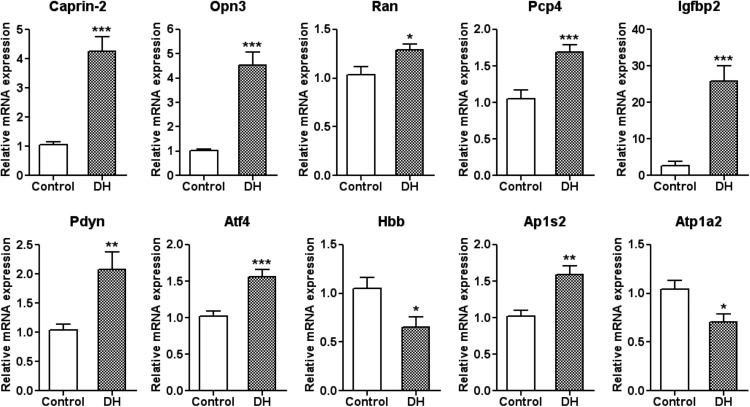
qRT-PCR validation of differential expression. The expression profiles of the 14 genes identified by Glasso were examined by qRT-PCR of RNA extracted from euhydrated (control) and dehydrated (DH) 12-week-old male SD SON. Of the original 14 genes in the Glasso network (Fig. 2*A*) 10 had significantly different relative expression levels. Error bar, SEM; *p < 0.05; **p < 0.01; ***p < 0.001; *n* = 10 for control and *n* = 11 for DH.

**Figure 4.**
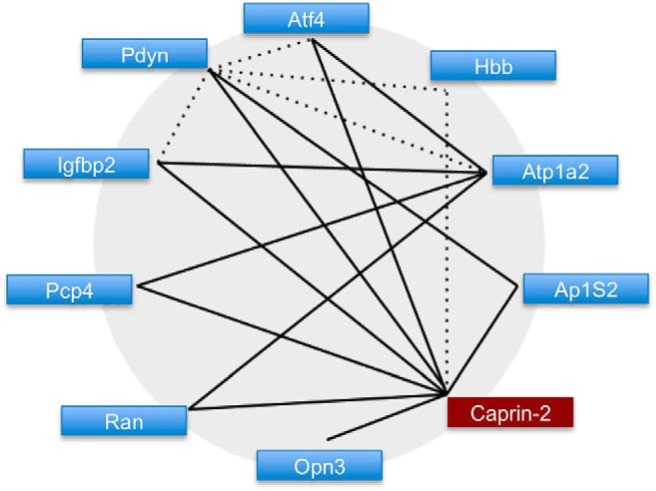
The validated Glasso-derived gene network. Link with significant Pearson correlation (*p* < 0.05) are represented by solid lines. Links not significantly correlated are represented by dotted lines.

We validated the expression profiles of the 14 genes identified by Glasso using qRT-PCR of RNA extracted from euhydrated and dehydrated 12-week-old male SD SON (*n* = 10 for euhydrated and *n* = 11 for dehydrated). Of the original 14 genes in the Glasso network (Fig. 2*A*), 10 had significantly different (*p* < 0.05) relative expression levels (Fig. 3), while three of the predicted genes were false positives and were therefore excluded (*Hmgn2*, BM391736; *Gja1*, AI411352; *Hba1*, AI179404). Primer sets for one of the expressed sequence tags (ESTs) AI577319 failed to deliver data. To establish whether correlations exist between the genes validated by qRT-PCR a Pearson correlation was performed which resulted in 8 out of 10 correlations (Fig. 4).

### Functional validation of the Glasso network *in vitro*

**Figure 5.**
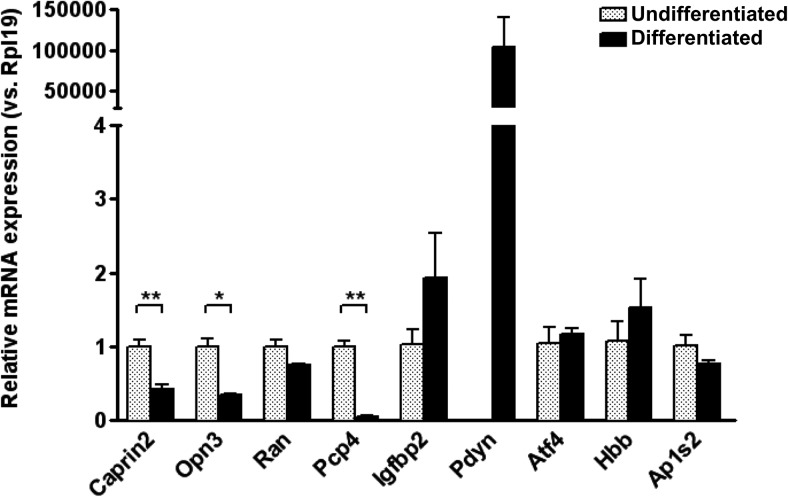
Expression of Glasso network genes in PC12 cells. Relative mRNA expression of genes in the network was examined in both undifferentiated and NGF-treated PC12 cells by qRT-PCR. The relative mRNA level was calculated using 2^(−ΔΔct) method where the expression level of undifferentiated sample was calculated as 1. Since *Pdyn* is undetectable at 40 cycles on qRT-PCR in undifferentiated PC12 cells, the relative mRNA level was calculated by assumption of Ct value of undifferentiated sample as 40 cycles. Error bar, SEM; *p < 0.05; **p < 0.01; unpaired Student’s *t* test; *n* = 3.

We developed an *in vitro* model to assess the functional validity of the Glasso predicted network. Rat adrenal pheochromocytoma PC12 cells can be made to differentiate into cells with a neurone-like phenotype by treatment with nerve growth factor (NGF). First, we used qRT-PCR to show that transcripts encoded by a sub-set of the genes of the Glasso network are expressed in PC12 cells, and that their expression level is changed following NGF treatment (Fig. 5). This analysis revealed that all the genes in the network, except Prodynorphin (*Pdyn*) and ATPase Na+/K^+^ transporting subunit-α2 (*Atp1a2*), are expressed in undifferentiated PC12 cells. Expression of *Pdyn* mRNAs are dramatically increased in NGF-treated cells (>100,000-fold increase), while the level of *Atp1a2* remains undetectable. The expression of *Caprin2*, Opsin-3 (*Opn3*), and Purkinje cell protein 4 (*Pcp4*) transcripts is decreased in PC12 cells following NGF treatment (*Caprin2* 0.425, *p* = 0.006; *Opn3* 0.343, *p* = 0.017; *Pcp4* 0.052, *p* = 0.003), while Ras oncogene family member *Ran*, insulin-like growth factor-binding protein 2 (*Igfbp2*), activating transcription factor 4 (*Atf4*), hemoglobin subunit-β (*Hbb*) and adaptor-related protein complex 1 σ2-subunit (*Ap1s2*) mRNA abundance are not significantly changed.

**Figure 6.**
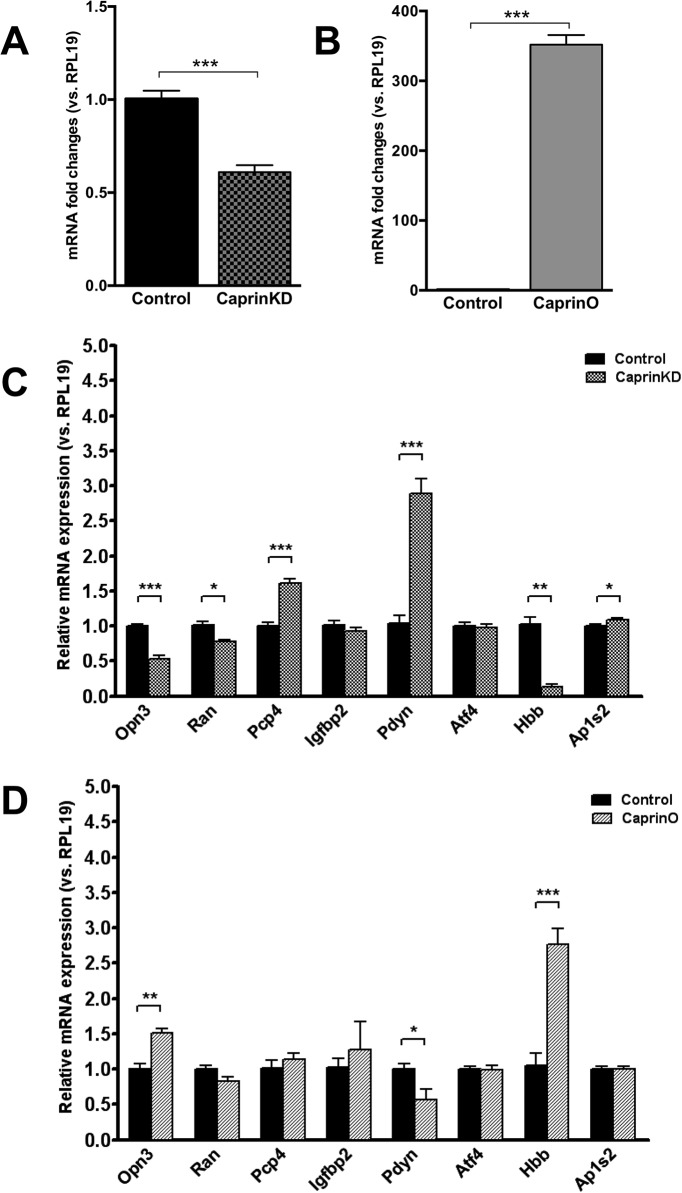
Change in mRNA expression of Glasso network genes in differentiated PC12 cells following *Caprin2* knockdown or *Caprin2* overexpression. ***A***, For knockdowm, NGF-treated PC12 cells were transduced with lentiviral vectors expressing either a Caprin2 shRNA (CaprinKD) or a scrambled shRNA (control). Relative mRNA expression of endogenous *Caprin2* was examined by qRT-PCR. The relative mRNA level was calculated using 2^(−ΔΔct) method, where the expression level of control sample was calculated as 1. Error bar, SEM; ***p < 0.001; unpaired Student’s *t* test; *n* = 6. ***B*,** For overexpression, NGF-treated PC12 cells were transduced with adenoviral vectors expressing either *Caprin2* (CaprinO) or *eGFP* (control). Relative mRNA expression of endogenous *Caprin2* was examined by qRT-PCR. The relative mRNA level was calculated using 2^(−ΔΔct) method where the expression level of control sample was calculated as 1. Error bar, SEM; ***p < 0.001; unpaired Student’s *t* test; *n* = 4. ***C***, NGF-treated PC12 cells were transduced with lentiviral vectors expressing either a *Caprin2* shRNA (CaprinKD) or a scrambled shRNA (control). Relative mRNA expression of genes in the network was examined by qRT-PCR. The relative mRNA level was calculated using 2^(−ΔΔct) method where the expression level of control sample was calculated as 1. Error bar, SEM; *p < 0.05; **p < 0.01; ***p < 0.001; unpaired *t* test; *n* = 6. ***D*,** NGF-treated PC12 cells were transduced with adenoviral vectors expressing either *Caprin2* (CaprinO) or *eGFP* (control). Relative mRNA expression of genes in the network was examined by qRT-PCR. The relative mRNA level was calculated using 2^(−ΔΔct) method where the expression level of control sample was calculated as 1. Error bar, SEM; *p < 0.05; **p < 0.01; ***p < 0.001; unpaired Student’s *t* test; *n* = 4.

We then manipulated the expression of nodal gene *Caprin2* in differentiated PC12 cells using lentiviral vectors that express either an shRNA specific for all splice isoforms of *Caprin2* (Konopacka et al., 2015) to knockdown endogenous expression (Fig. 6*A*), or an Adenoviral vector expressing full-length rat *Caprin2* cDNA (Konopacka et al., 2015) to elicit overexpression (Fig. 6*B*). We then used qRT-PCR to ask about the effects of *Caprin2* knockdown (Fig. 6*C*) or overexpression (Fig. 6*D*) on the levels of transcripts encoded by putative target genes within the Glasso network. With the exception of the *Atf4* and *Igfbp2* mRNAs, the expression of which was not altered by manipulation of *Caprin2*, knockdown or overexpression of *Caprin2* *in vitro* had opposite effects on *Pdyn*, *Opn3* and *Hbb* mRNA abundance. Thus, while Caprin2 knockdown increased Pdyn mRNA levels (3.833, *p* = 0.00004), *Caprin2* overexpression decreased *Pdyn* transcript abundance (0.572, *p* = 0.035). In contrast, both *Opn3* and *Hbb* levels were decreased by *Caprin2* knockdown (*Opn3* 0.477, *p* = 0.00004; *Hbb* 0.323, *p* = 0.0013), but were increased following *Caprin2* overexpression (*Opn3* 1.506, *p* = 0.0023; *Hbb* 2.771, *p* = 0.0008).

## Discussion

Following stimulatory physiologic cues, such as dehydration, salt-loading and lactation, the SON of the hypothalamus undergoes a function related plasticity that has previously been described in the rat at the transcriptome level (Hindmarch et al., 2006; Qiu et al., 2011; Greenwood et al., 2015). Using the unsupervised Glasso algorithm (Friedman et al., 2008), we sought to reconstruct a putative network from 500 plastic SON genes in which genes are the nodes and the edges are the inferred interactions. The most active nodal gene identified within the network was RNA-binding protein *Caprin2*. To test the validity of the Glasso approach, we sought an *in vitro* cell culture model in which genes of the predicted network are expressed. We found that differentiated PC12 cells met these criteria. We then either overexpressed or knocked down *Caprin2* transcripts in differentiated rat pheochromocytoma PC12 cells, and showed that these manipulations had significant opposite effects on the levels of putative target mRNAs.

A major bottleneck of the “omic” era is the sheer scale and complexity of the datasets, and the resulting daunting problem of identifying suitable targets for often expensive and time consuming physiologic studies. We sought to address this problem in a mammalian homeostatic system that demonstrates functionally important plasticity following the physiologic challenges of dehydration, salt-loading and lactation. Each of these physiologic challenges activates the SON in a similar manner resulting in a function related plasticity that facilitates an appropriate neuroendocrine response. We hypothesized that there is a common network of genes in this tissue that underpins the general plasticity of the SON. to test this hypothesis, we combined data from the activated plastic SON, e.g., male and female water deprived, male salt-loaded and female lactation, and compared this to the male and female “naïve” data. This comparison resulted in a list of significantly regulated genes on which we performed unsupervised network inference using Glasso to reconstruct a gene network from the SON of the rat hypothalamus in either naïve or physiologically stimulated states, thus allowing us to identify potentially important “hub” genes with high numbers of putative regulatory links (i.e., they have a large fan-out in circo graphs) that may have prominent functional roles. Network inference, while less accurate than supervised methods, is a useful first step in the absence of a training set of known links and non-links. Rather than performing large numbers of perturbation experiments to characterize the entire network, we can use existing microarray data to generate correlations between genes and by applying network inference predict a of number high confidence links, giving us target genes for perturbation experiments, hence maximizing our efforts.

The application of Glasso resulted is a network of 28 genes with 48 links, while Pearson placed 47 genes in a network of 32 links. Only one gene, *Caprin2*, appeared in both networks. To mitigate the false discovery rate further, the network was reduced by eliminating genes below a “richness” criterion. Only those genes that the network infers as having links to at least 2 genes, and that both those genes should be linked to at least two others, were included (Fig. 2). Candidate genes identified from the Glasso inference were then validated by qRT-PCR of SON RNA collected from either euhydrated or dehydrated male rats (Fig. 3). Following elimination of the three genes that did not validate, as well as one that did not deliver data, the remaining genes were subject to Pearson correlation to attempt reengineering of the network (Fig. 4). Examination of this network clearly reveals a central hub gene, *Caprin2*, an RNA-binding protein (Shiina and Tokunaga, 2010) that has been shown to bind the *AVP* mRNA (Konopacka et al., 2015) and to mediate changes in *AVP* mRNA abundance and poly(A) tail length (Konopacka et al., 2015). Lentiviral mediated shRNA knockdown of *Caprin2* in the osmotically stimulated hypothalamus shortened the *AVP* mRNA poly(A) tail and reduced transcript abundance (Konopacka et al., 2015). In an *in vitro* system, *Caprin2* overexpression enhanced the abundance and poly(A) tail length of the *AVP* mRNA (Konopacka et al., 2015).

To test the physiologic validity of the Glasso network centered on *Caprin2*, we developed an *in vitro* system that enabled us manipulate *Caprin2* expression and ask about consequential effects on the steady-state levels of putative transcript targets. As the network is based on transctriptome data, the functional links must regulate mRNA abundance. First, we showed that NGF differentiated PC12 cells express all of the genes in the network, except for one (*Atp1a2*; Fig. 5). We then used viral-mediate gene transfer to either knockdown *Caprin2* using a previously characterized specific shRNA (Konopacka et al., 2015), or to overexpress *Caprin2* (Fig. 6). As a consequence, we saw opposite effects on the levels of putative target mRNAs [knockdown (Fig. 6*C*), overexpression (Fig. 6*D*)]. Thus, while *Caprin2* knockdown decreased the abundance of *Opn3*, *Ran* and *Hbb* transcripts, overexpression increased the levels of *Opn3* and *Hbb* RNAs. In contrast, *Caprin2* knockdown increased *Pcp4*, *Pdyn*, and *Ap1s2* RNAs, while overexpression decreased Pdyn transcript abundance. *Igfbp2* and *Atf4* RNA levels were unaffected by Caprin2 manipulation.

In the dehydrated SON, an increase in *Caprin2* mRNA expression is accompanied by an increase in the abundance of *Opn3*, *Ran*, *Pcp4*, *Igfbp2*, *Pdyn*, *Atf4*, and *Ap1s2* transcripts, but a decrease in *Hbb* mRNA abundance. Consistent with this, the decrease in *Caprin2* expression following differentiation of PC12 cells reduces *Opn3* and *Pcp4* transcript levels. However, *Pdyn* levels dramatically increase as a consequence of NGF-mediated differentiation of PC12, probably as a consequence of a separate *Caprin2*-independent transcriptional mechanism. We then manipulated *Caprin2* activity in differentiated PC12 cells by overexpression or shRNA-mediated knockdown to ask about effects on the expression on putative network interacting genes. In some cases, we saw dramatic opposite effects. Thus, *Caprin2* knockdown reduced *Opn3* and *Hbb* mRNA levels, but increased *Pdyn* transcript abundance, whereas *Caprin2* overexpression increased *Opn3* and *Hbb* mRNA levels, but reduced *Pdyn* transcript abundance. These data suggest that *Caprin2* functions to increase *Opn3* and *Hbb* expression, but to decrease *Pdyn* expression. These data are consistent with the increased *Opn3* mRNA abundance seen in the dehydrated SON, perhaps through direct association and consequent stabilisation. However, this is contrary to expectation in terms of *Pdyn*, which has increased expression in the dehydrated SON, and *Hbb*, which has decreased expression in the dehydrated SON. We suggest that any role for *Caprin2* in *Pdyn* and *Hbb* mRNA regulation may not be direct and need to be considered in the context of other, possibly transcriptional, mechanisms.

Our data raise questions regarding the molecular nature and physiologic consequences of the regulatory interactions in the *Caprin2* gene network. *Caprin2* knockdown in the *in vivo* hypothalamus leads to dysfunction of the normal physiologic response to salt loading, an osmotic challenge, which in healthy rats leads to a gradual increase of urine output and fluid intake. *Caprin2* knockdown results in a significant decrease in urine output and fluid intake, and an increase in urine osmolality and plasma AVP levels (Konopacka et al., 2015). We hypothesize that these dramatic physiologic consequences are a sum-total of changes in the expression of the gene products encoded by *Caprin2* target genes. That Pdyn is a putative target of *Caprin2* is instructive in this regard. The expression of the endogenous opioid peptide *Pdyn* within the hypothalamus is well known, as is its upregulation following dehydration (Sherman et al., 1986). Dynorphin peptide colocalizes with AVP, and both can be released either from axons or somato-dendritically. At the level of the neural lobe, dynorphin is coreleased with AVP and acts on axon terminal κ−opiate receptors to inhibit electrically evoked secretion of oxytocin (Falke, 1988). Centrally, dendritic release of dynorphin appears to regulate MCN electrical activity (Brown and Bourque, 2004; Brown et al., 2004; Brown et al., 2006). It is thus possible that *Caprin2* mediates some of its physiologic effects through the actions of dynorphin. Interestingly, the *Hbb* gene is expressed in the brain (Ohyagi et al., 1994), and encodes the hemorphins, a family of endogenous nonclassical opioid peptides (Nyberg et al., 1997; Zhao et al., 1997).

Using transcriptome data from the physiologically plastic SON as a model, we have shown that our unbiased network inference strategy, using Glasso, has predictive value, and can “enrich” for functional interactions that can be tested experimentally. The application of Glasso to extensive transcriptome datasets will accelerate the identification of physiologically relevant pathways.

## Synthesis

Reviewing Editor: Quentin Pittman, University of Calgary Decisions are customarily a result of the Reviewing Editor and the peer reviewers coming together and discussing their recommendations until a consensus is reached. When revisions are invited, a fact-based synthesis statement explaining their decision and outlining what is needed to prepare a revision will be listed below. The following reviewer(s) agreed to reveal their identity: Roman Romanov.

Major points for revision:

1. While the reviewers agree that your manuscript presents an interesting study, they both believe that in its present form the manuscript is quite difficult to understand and not approachable for a non-mathematician. Thus they would like to see you edit the manuscript, possibly with help from a colleague not immersed in statistical analysis approaches to make it more accessible to the reader, as well as to highlight the physiological relevance of the data.

2. Both reviewers question the use of PC12 cell lines rather than cultured SON neurons; while they both understand that the data being what they are, some justification should be in place to discuss this matter.

3. The non-physiological 72h dehydration was a surprising approach and relevance to more reasonable stressors on the system should be addressed.

4. A number of other more minor issues were raised and these can be seen in the two reviews which are appended below:

Reviewer 1:

While the manuscript is of wide interest for bioinformaticians and neuroscientists, there are some questions are still left and corrections are needed to be done.

There is quite complicated mathematical basis for the experimental procedures, which needs time to understand. So using of unconventional formulas symbols makes it harder and takes much more time (however the authors starts to use right symbols in the discussion). The same is right for gene code number identifiers presented in tables. Please use also short gene names everywhere to be readable.

It would be also quite helpful to make the part about conditional number mu (page 8) more easy to understand since the reason of choosing the value for it is not clear. What does it mean? How was it calculated? The ref. Cheney and Kincaid was not so helpful.

Likewise, there are some mathematical and computational approaches, which seem to be overcomplicated. For instance, why Kullback-Leibler divergence was used? The authors claimed that standard deviation was in a range of 10 %. Meantime, Laurens van der Maaten (Journal of Machine Learning Research 1 (2008) 1-48) wrote that selection of this parameter is optimal for the dataset if the density of the data is likely to vary.

Next, for the data presented in the figure 2 it would be good to see the real numbers (correlation coefficients etc) for both of methods (may be as a comparative table).

Finally, the results shown at figures 4 and 5 rise questions. Results look to be satisfactory validation in vitro. However, the small changes of carpin level in KD experiments and a huge effect on carpin expression level in the overexpression model led to a feeling that control levels of carpin are very low in the PC12 cells. Thus, it is questionable whether the in vitro model is adequate enough. I think a little bit more information about the base levels, transfection efficacy would be helpful.

Minor: The figure 2 c should go after validation experiments.

Reviewer 2:

The authors report novel data concerning a hub role for the gene Caprin2 in the plasticity of neurosecretory neurons with hormone release challenge. Caprin2 is an mRNA binding protein that affects vasopressin as well as other mRNAs, and which appears involved in conditions where both oxytocin and vasopressin release are increased, and hormone production is upregulated. The authors have re-analyzed existing microarray data from a variety of experimental conditions (dehydration, lactation, salt challenge) for network construction using two independent statistical methods, and find Caprin2 the only gene changed in these states that appears in both the Glasso and Pearson correlation methods. They then further identified 14 genes of interest that are modified with severe dehydration using PCR. Most of these (8) were further analyzed after manipulating Caprin2 expression in PC12 cells, and 5-6 of them were affected by either knock down or overexpression of Caprin2.

While uncovering effects of Caprin2 manipulation in PC12 cells, the authors did point out that the direction of effects did not match the expected association of Caprin2, Hbb and Pdyn, the two latter genes being affected by Caprin2 manipulation in ways opposite of their prediction from the PCR data on dehydrated rats. This suggests a much more complicated, and probably indirect role, for Caprin2's modulation of Hbb and Pdyn during these challenges.

The paper is well done and despite the somewhat overwhelming and jargon-laden description of the statistical analyses, one is confident that care has been taken with the data and this funneling toward the fewer players of interest has real merit. I have the following issues that should be addressed:

1) Why didn't the authors investigate effects of Caprin2 expression on cultured SON, instead of PC12, neurons? I am not sure of the significance of the PC12 data, with and without NGF, regarding their purpose in studying homeostasis in magnocellular neurons. I understand they are models of some sort, but why not go directly to cells of interest, as they are easily cultured and are the putative targets of their long term studies. Alternatively, they could have transfected PC12 cells with VP or OT mRNA for this type of study.

2) On a related point, they have shown that Caprin2 binds to VP mRNA and regulates its expression. However, neither VP mRNA, nor OT mRNA emerged from their data set of important “plasticity” factors in the microarray data, despite both are known to be dramatically upregulated by these stimuli. Isn't this surprising? This bears discussion since they are using Caprin2's effects on VP mRNA as a measure of its importance in plasticity in these neurons.

3) Is there significance to Caprin2 ranking 50th overall in a t-test from the Glasso reconstruction? In Figure 1, I would mark the crossover region where meaningful data is distinguished from random in 1A. In Figure 1B, the random data appears as an easily missed vertical line the upper left- this should be noted in the caption since it's not obvious and it clearly distinguishes random from the significant data.

4) Could the authors be more specific about the 3 false positives and the primer which gave no data, using gene names rather than accession numbers? I would list these in Table 1 as well since they were tested

5) The Discussion is a bit long and somewhat repetitive regarding the statistical approaches used. While I understand this analysis was the main point of the paper, I would still cut back areas that report methods for example, and get to the important points quicker- meaning, interpreting the importance of the genes they show are regulated by Caprin2 as for as supraoptic neurons are concerned.

6) The authors use 72 hr dehydration, which is non-physiological and right on the cusp of being deleterious to the animal. This amount of dehydration is associated with an 80-90% drop in food consumed for example (which means a much more complex stimulus), and may reduce blood volume more significantly that it increases osmolality. This stimulus should be justified, because in the course of a normal rat's (or human's) life, probably wouldn't occur, and thus loses some of its interest for that reason. Also, why did they only choose this measure for the PCR studies, and not also lactation as was used in the microarrays? Wouldn't lactation be expected to give a different set of genes given its focus on oxytocin? Not to mention, its more physiological. For females.

Minor

1) As they did in the last figure, I would re-color Caprin2 in Figure 2.

2) Page 13, line 13- there is no reference to the abbreviation EST

3) Page 12, line 7- “......three physiological transitions”, not “transition”

4) Abstract, line 10 “....algorithm is that it can...”, not “is it can”
